# Effects of organic and inorganic copper on cecal microbiota and short-chain fatty acids in growing rabbits

**DOI:** 10.3389/fvets.2023.1179374

**Published:** 2023-05-19

**Authors:** Yanan Du, Yun Tu, Zeyang Zhou, Rui Hong, Jiayou Yan, Gong-Wei Zhang

**Affiliations:** ^1^College of Animal Science and Technology, Southwest University, Chongqing, China; ^2^Animal Breeding and Genetics Key Laboratory of Sichuan Province, Sichuan Animal Science Academy, Chengdu, China

**Keywords:** gut microbiome, organic copper, rabbits, SCFAs, cupric citrate

## Abstract

**Introduction:**

Copper (Cu) is an essential trace element for the growth of rabbits. This study aimed to investigate the effects of different Cu sources on intestinal microorganisms and short-chain fatty acids (SCFAs) in growing rabbits.

**Methods:**

The experimental animals were randomly divided into four experimental groups, each group comprised eight replicates, with six rabbits (half male and half female) per replicate. And they were fed diets was composed by mixing the basal diet with 20 mg/kg Cu from one of the two inorganic Cu (cupric sulfate and dicopper chloride trihydroxide) or two organic Cu (cupric citrate and copper glycinate). Cecal contents of four rabbits were collected from four experimental groups for 16S rDNA gene amplification sequencing and gas chromatography analysis.

**Results:**

Our results indicate that the organic Cu groups were less variable than the inorganic Cu groups. Compared with the inorganic Cu groups, the CuCit group had a significantly higher relative abundance of *Rikenella Tissierella*, *Lachnospiraceae_NK3A20_group*, *Enterococcus*, and *Paeniclostridium*, while the relative abundance of *Novosphingobium* and *Ruminococcus* were significantly lower (*p* < 0.05). The SCFAs level decreased in the organic Cu groups than in the inorganic Cu groups. Among the SCFAs, the butyric acid level significantly decreased in the CuCit group than in the CuSO_4_ and CuCl_2_ groups. The relative abundance of *Rikenella* and *Turicibacter* genera was significantly negatively correlated with the butyric acid level in the CuCit group compared with both inorganic Cu groups. These results revealed that the organic Cu (CuCit) group had an increased abundance of *Rikenella*, *Enterococcus*, *Lachnospiraceae_NK3A20_group*, and *Turicibacter* genera in the rabbit cecum.

**Discussion:**

In summary, this study found that organic Cu and inorganic Cu sources had different effects on cecal microbiota composition and SCFAs in rabbits. The CuCit group had the unique higher relative abundance of genera *Rikenella* and *Lachnospiraceae_NK3A20_group*, which might be beneficial to the lower incidence of diarrhea in rabbits.

## 1. Introduction

Cu is routinely supplemented to animal diets at concentrations above the nutritional requirement of the animals because the pharmacological concentrations of inorganic cupric sulfate (CuSO_4_) have been shown to have growth-stimulatory properties, for example, 125 or 250 mg/kg of Cu in the broiler diet (1) and 242 mg/kg of Cu in the pig diet (2). However, the pharmacological concentrations of dietary Cu present an environmental concern because of its high excretion in feces (3). More studies revealed that organic Cu additive might be more environmentally friendly than CuSO_4_ supplements, which could attenuate fecal acidity, diarrhea incidence, and fecal Cu concentration (4, 5). Cupric citrate (CuCit), an organic form of Cu, could stimulate growth at lower concentrations than CuSO_4_ in broiler chickens (1, 6) and weanling pigs (4, 7). In China, the maximum amount of Cu in meat rabbit formula feed or fully mixed diet is 25 mg/kg (including the background value of feed raw materials). Previous studies revealed that CuCit had a beneficial effect on the average daily gain and feed weight ratio than CuSO_4_ at the same dose (20 mg/kg of Cu) in the rabbit diet (8). Interestingly, the incidence of diarrhea was reduced when the rabbits were fed with CuCit diets compared with other Cu source diets. The incidence of diarrhea in the CuCit group was 8.3%, which was 16.7–18.7% in the other Cu source groups (8). However, studies exploring the mechanisms by which low dietary CuCit reduced the incidence of diarrhea in rabbits were few.

One of the possible mechanisms by which Cu may benefit animals was by shifting the gastrointestinal microbiota, thereby reducing the susceptibility of animals to diseases (9). Numerous studies confirmed the various properties of Cu as antibacterial, antifungal, and antiviral agents (10). Different sources of Cu have different effects on the intestinal structure and microflora (4). A pharmacological dose of Cu significantly affected the composition of microbial communities in the ileum and cecum of weaned piglets (11). The high level of Cu significantly reduced the abundance of enterococci and lactic acid bacteria (12) or lactobacilli (13) in the porcine gut. The supplementation with 36.75 mg/kg of Cu from Cu-bearing montmorillonite reduced the total viable counts of *Escherichia coli* and *Clostridia* in the small intestine and cecum of broiler chicken (14). The abundance of *Rikenella* and *Barnesiella* genera increased in Sprague–Dawley rats in the 240 mg/kg Cu group compared with the 6 mg/kg Cu group (15). Meanwhile, the changes in the intestinal microbiota composition were shown to alter the abundance of bacterial metabolites (16, 17). Short-chain fatty acids (SCFAs), mainly acetate, propionate, and butyrate, are recognized as important metabolites derived from the fermentation of indigestible dietary fibers by gut microbial species, which play fundamental roles in maintaining intestinal homeostasis and regulating energy metabolism (18). These intestinal microbial communities played an important role in the growth and gut health of animals by producing SCFAs in chickens (19) and rabbits (20). The SCFAs were the primary energy sources of the colonic epithelial cells in mice (21). Therefore, this study used 16S rDNA gene amplicon sequencing and gas chromatography, respectively，aimed to evaluate the impact of dietary supplementation of four different Cu sources at the same level on cecal microbiota and SCFAs in growing rabbits.

## 2. Materials and methods

### 2.1. Materials

In this study, cupric citrate (CuCit) was provided by Sichuan Animtech Feed Co., Ltd., Chengdu, China. CuCit is a kind of organic Cu, with a purity of approximately 98.5%, and the content of Cu was 34.5%. Cupric sulfate (CuSO_4_·5H_2_O, Cu > 25.1%, abbreviated as CuSO_4_), dicopper chloride trihydroxide (Cu_2_(OH)_3_Cl, Cu > 58.1%, abbreviated as CuCl_2_), and copper glycinate (CuGly, Cu > 21%) were bought from the local market.

### 2.2. Collection of the cecal contents in rabbits

The animal care and experimental design were conducted as described in a previous study (8). In brief, 240 New Zealand rabbits, aged 35 days, were randomly divided into four experimental groups and one control group, each group comprised 8 replicates, with 6 rabbits (half male and half female) per replicate. The basal diet was formulated to meet the requirements for growing rabbits, the digestive energy was 10.08 MJ/kg, the crude protein level was 15.85%, and the crude fiber level was 15.08%. The basel diet added copper as the control group which contained 5.00 mg/kg Cu from the raw materials (Table 1). Four treatment groups were added the 20 mg/kg Cu from CuSO_4_, CuCl_2_, CuCit, and CuGly to the basel diet, respectively. Thus, the total copper content in these four experimental diets was 25 mg/kg. These animals were treated with the same nutritional management. The rabbits were raised for 49 days after 7 days of adaptation. At the end of the experiment, four rabbits were slaughtered from each group. The entire cecal contents were first blended sufficiently and then collected from each rabbit. In this study, only the cecal samples from the four Cu experimental groups were selected for 16S rDNA gene amplicon sequencing and gas chromatography. All procedures were approved by the Institutional Animal Care and Use Committee of the Sichuan Academy of Animal Science.

**Table 1:** Diet composition and nutritional composition (air-dried base).

Project	Content
*Raw material composition, %*	
Alfalfa grass powder	30.00
Maize	17.00
Bran	22.50
Secondary powder	5.00
Commanding chaff	7.00
Soybean meal (43% CP)	8.50
Rapeseed meal	4.60
Puffed soybeans	2.00
Calcium carbonate	1.00
Dicalcium phosphate	0.70
L–Lysine hydrochloride	0.26
DL-methionine	0.04
Table salt	0.40
Premixed feed^a^	1.00
Total	100.00
*Nutrition facts* ^b^	
Digestive energy, MJ/kg	10.08
Crude protein, %	15.85
Crude fiber, %	15.08
Calcium, %	1.08
Phosphorus, %	0.65
Lysine, %	0.92
Methionine + cystine, %	0.64
Copper, mg/kg	5.00

a Premixed feed provides per kilogram of diet: vitamin A 10,000 IU, vitamin D3 1,000 IU, vitamin E 20 IU, vitamin K_3_ 1 mg, vitamin B_1_ 2 mg, vitamin B_2_ 2 mg, vitamin B_6_ 1 mg, vitamin B_12_ 10 μg, niacin 50 mg, calcium pantothenate 20 mg, folic acid 0.1 mg, Biotin 0.2 mg, choline 300 mg, iron 50 mg, zinc 80 mg, manganese 8.5 mg, selenium 0.05 mg, iodine 0.2 mg.

b The digestion energy in the nutrient composition is the calculated value, and the rest are measured values.

### 2.3. DNA extraction, polymerase chain reaction amplification, and sequencing

Cecal microbial genomic DNA was extracted using a Magnetic Soil and Stool DNA Kit (Qiagen, CA, United States) following the kit instructions. DNA concentration and purity were determined using a NanoDrop 2000 ultraviolet–visible spectrophotometer (Thermo Scientific, DE, United States). The primers of 341F (5′-CCTAYGGGRBGCASCAG-3′) and 806R (5′-GGACTACNNGGGTATCTAAT-3′) with the barcode were used to amply the highly variable regions V3–V4 of the bacterial 16S ribosomal RNA (rRNA) gene. The polymerase chain reaction (PCR) was carried out using the Phusion High-Fidelity PCR Master Mix (New England Biolabs), and the PCR products were purified using the Qiagen gel extraction kit (Qiagen, Germany). The sequencing libraries were generated using the TruSeq DNA PCR-free sample preparation kit (Illumina, CA, United States). Purified amplicons were paired-end sequenced on an Illumina NovaSeq 6000 platform (Illumina, CA, United States).

### 2.4. Bioinformatics analysis of sequencing data

The paired-end reads were assigned to samples based on their unique barcodes and primer sequences and merged using FLASH (v.1.2.7, http://ccb.jhu.edu/software/FLASH/). Quantitative Insights Into Microbial Ecology (QIIME, v.1.9.1, http://qiime.org/scripts/split_libraries_fastq.html) was used for the quality control of sequencing data to obtain high-quality clean tags. Operational taxonomic units (OTUs) (*de novo*) were picked using UPARSE (v7.0.1001, http://www.drive5.com/uparse/) with a 97% similarity threshold. Alpha diversity, as indicated by Observed-OTUs, Chao1, Simpson, and Shannon index, was calculated. Analyses of similarities (ANOSIM and Adonis) were used for determining the significance analysis of beta diversity. MetaStats (set to 5,000 permutations for the nonparametric *t*-test) and *t*-test were employed for differential abundance analysis between groups. The R (3.6.0) software was used to draw box plots and conduct principal components analysis (PCA), and nonmetric multidimensional scaling (NMDS).

### 2.5. SCFA extraction and analysis

SCFAs were extracted by Metware Biotechnology Co., Ltd. (Wuhan, China). Briefly, 20 mg of cecal contents were dissolved in 1 ml of phosphoric acid (0.5% v/v) solution, mixed by spinning, and sonicated in an ice bath for 5 min. Then, the solution was centrifuged at 12,000 rpm for 10 min at 4°C. Further, 100 μl of the supernatant was taken, and 500 μl of Methyl tert-butyl ether (MTBE) solvent containing internal standards was added and vortexed for 3 min. After sonication in an ice bath for 5 min, the mixture was centrifuged at 12,000 rpm for 10 min at 4°C. After centrifugation, 200 μl of the supernatant was pipetted off for gas chromatography–tandem mass spectrometry analysis. The analysis of variance was performed to detect the differences in the levels of SCFAs in the cecum.

### 2.6. Microbial combined analysis with SCFAs

Microbial and SCFA data were unit variance scaled. Spearman correlation analysis between microbial and SCFA data was calculated using the cor function of the R software, and significance tests for correlation were conducted using the corPvalueStudent function of the Weighted correlation network analysis (WGCNA) package of the R software. Spearman correlation with |*r*| ≥ 0.8 and *p* value < 0.05 were considered to have a significant correlation coefficient. The R (3.6.0) software was used to draw the Spearman circos and correlation scatterplot.

## 3. Results

### 3.1. Microbial community composition and microbiome diversity in the cecum of the groups fed with four different Cu sources

**Figure 1:**
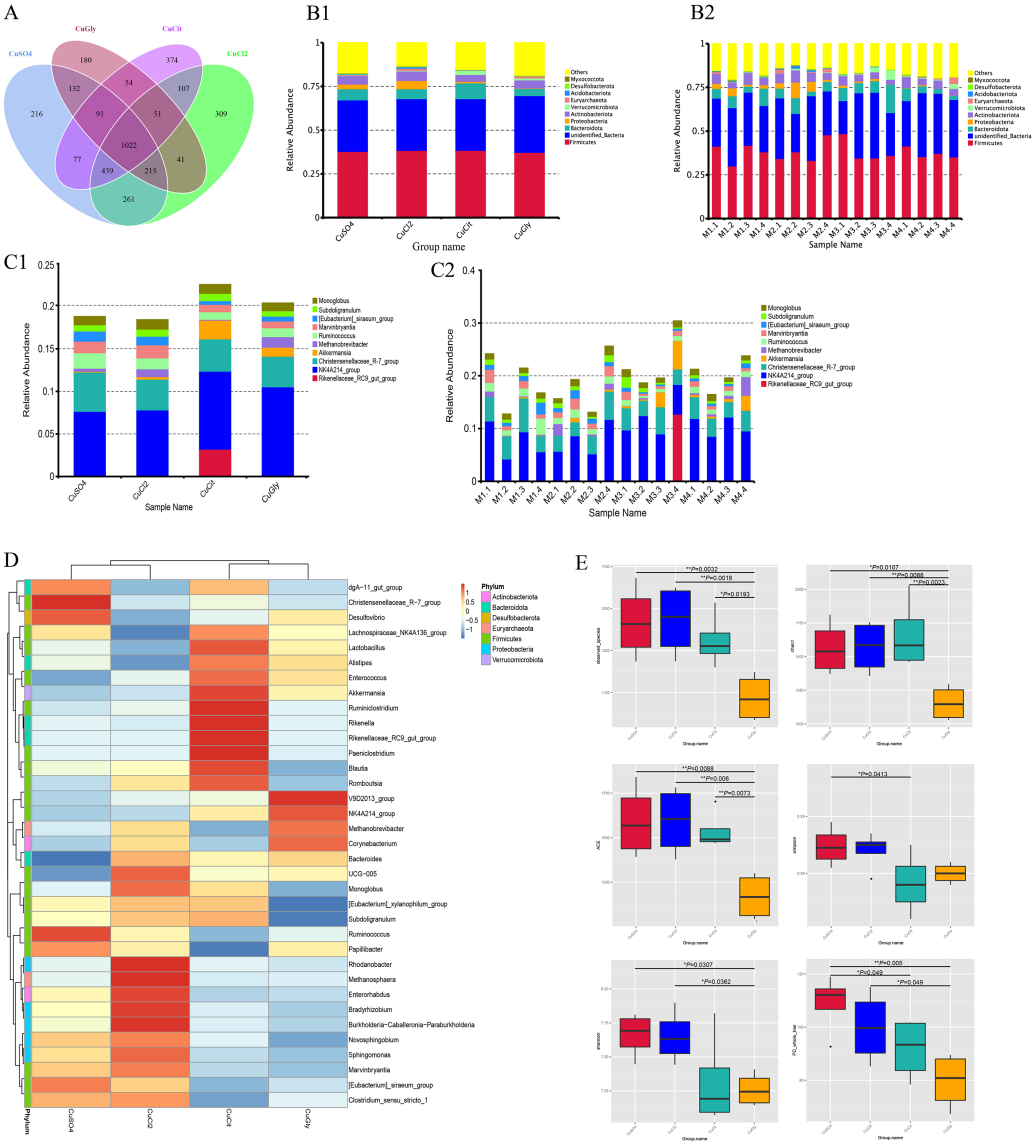
Cecal microbial community composition and alpha diversity from four different copper sources in rabbit diet. **(A)** Venn plot of the OTU. **(B)** Relative abundance of the top 10 bacterial phyla for each group **(B1)** and per individual **(B2)**. **(C)** Relative abundance of the top 10 bacterial genera in each group **(C1)** and per individual **(C2)**. **(D)** Cluster heatmap of top 35 genera. **(E)** Box plot for the analysis of differences between groups in alpha diversity index.

A total of 1,360,930 reads with an average length of 407 bp per read were obtained after quality filtering using the UPARSE software. A total of 3,569 OTUs were identified at the 97% similarity level using these filtered sequences. Among these, 1,022 OTUs were common in the four groups, and the number of unique OTUs was higher in the CuCit organic Cu group than in the other groups (Figure 1A). These OTUs were taxonomically annotated to a total of 43 phyla, 102 classes, 239 orders, 333 families, 534 genera, and 224 species. The top 10 OTUs were composed of the microbial communities of cecal samples from the groups fed with four different Cu sources, and each individual at the phylum and genus levels are summarized in Figures 1B,C. As observed in all groups and individuals, the order of abundance at the phylum level was *Firmicutes* followed by *unidentified_Bacteria* and Bacteroidota (Figures 1B,1C1). The CuCit group had higher relative abundance of phyla Bacteroidota and Verrucomicrobiota compared with the other groups (Figure 1B). At the genus level, *NK4A214_group* was the most abundant, followed by the *Christensenellaceae_R-7_group* (Figures 1C,1C2). The *Rikenellaceae_RC9_gut_group* showed abundance in the cecum of one individual (M3.4), which led to the specifically higher relative abundance in the CuCit group (Figures 1C,1C2). The heatmap of 35 genera showed that the CuCit group had a specific higher relative abundance of *Rikenella* and *Paeniclostridium* genera (Figure 1D). The inorganic Cu groups had higher relative abundance of *Marvinbryantia* and *[Eubacterium]_siraeum_group* genera compared with the organic Cu groups (Figure 1D).

The richness and evenness information about the microbiome diversity across the four different Cu sources, and common and unique OTU information among different groups, were obtained using alpha diversity assessment. The CuGly treatment group showed significantly lower species richness in terms of the Observed-OTUs, Chao1, and Abundance-based Coverage Estimator (ACE) compared with the other three groups (*p* < 0.05) (Figure 1E). For the Shannon index, the CuGly were less variable than inorganic Cu groups CuCl_2_ and CuSO_4_ (*p* < 0.05) (Figure 1E). No obvious difference was found among the four groups in terms of the Simpson index (Figure 1E). These results revealed that these organic Cu groups had lower species richness compared with the inorganic Cu groups.

### 3.2. Different cu sources altered the microbiota composition in the rabbit cecum

**Figure 2:**
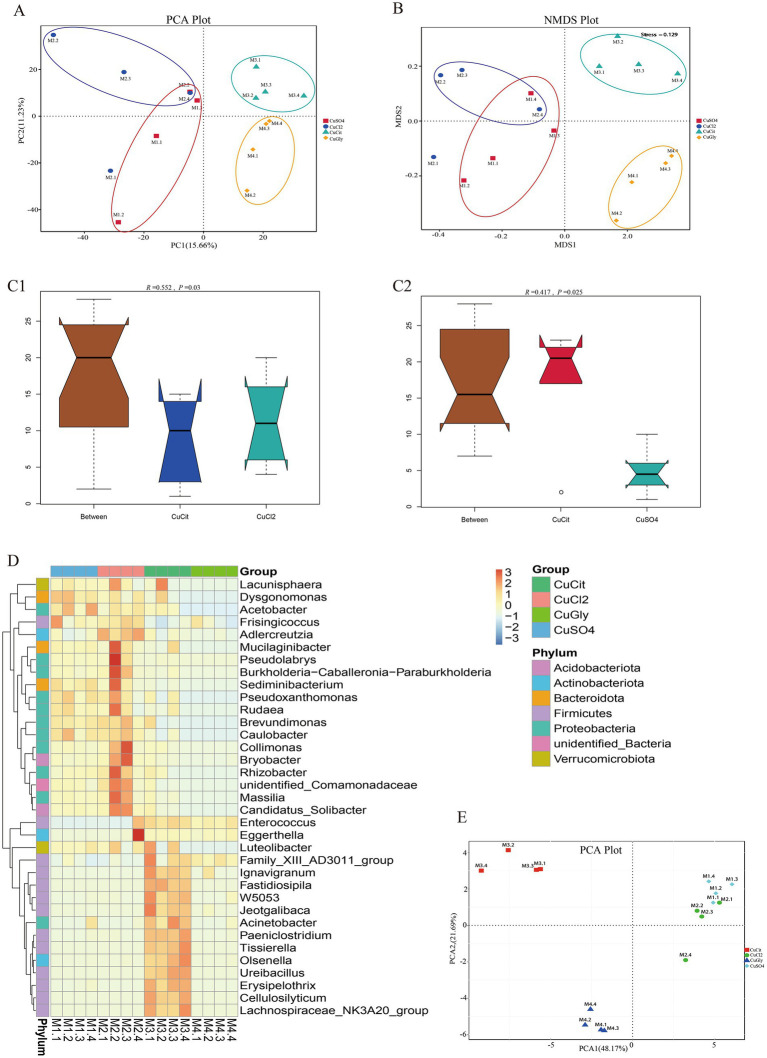
Different copper sources altered the microbiota composition in the rabbit cecum. **(A,B)** PCA score plot and NMDS score plot based on unweights. **(C1,C2)** ANOSIM plot of cupric citrate (CuCit) compared with two inorganic coppers. **(D)** MetaStat of cluster at the genus level. **(E)** PCA plot of top 35 genera from MetaStat analysis.

Beta diversity measures were assessed to capture the changes in the microbiome community composition among the groups. We first employed the PCA method for clustering all samples based on the OTU profile. These 16 samples were clearly clustered in the spaces of PC1 and PC2 (Figure 2A), respectively. The differences between organic and inorganic Cu groups could be explained by PC1, while the difference between two different sources of organic Cu or inorganic Cu was explained by PC2 (Figure 2A). The NMDS method also showed that these four groups were clustered in different quadrants (Figure 2B). These results indicated significant differences between organic and inorganic Cu groups.

The ANOSIM and Adonis analyses were used to learn more about the difference in the microbiome community among the four different Cu sources (Table 2). As a nonparametric test, ANOSIM could evaluate whether the variation in the cecal microbiome among the four different Cu sources was significantly larger than the variation within groups. This information helped us evaluate the reasonability of the division of groups. Our ANOSIM results showed a positive *R* value and *p* value < 0.05, which suggested that the intergroup variation was larger than the intragroup variation (Figure 2C; Table 2). Adonis is a nonparametric multivariate variance analysis method based on the Bray–Curtis distance. This method could analyze the interpretation of different grouping factors to sample differences. In total, ANOSIM and Adonis analyses suggested a significant difference between the organic and inorganic Cu groups at the OTU level (Table 2).

**Table 2:** ANOSIM/adonis analyses of microbial communities in different groups.

Item	ANOSIM		Adonis	
	*R*	*p*	*R* ^2^	*p*
CuCl_2_-CuCit	0.552	0.03	0.227	0.03
CuSO_4_-CuCit	0.417	0.025	0.228	0.001
CuSO_4_-CuCl_2_	−0.052	0.611	0.130	0.635
CuGly-CuCit	0.187	0.03	0.181	0.022
CuGly-CuCl_2_	0.364	0.026	0.246	0.001
CuGly-CuSO_4_	0.427	0.057	0.223	0.022

Several analytic methods were used to identify the specific clades of the microbiome in the four groups, including the MetaStat and the *t*-test analyses. In MetaStat analysis, *q* < 0.05 was confirmed as a significant difference. At the genus level, the cluster heatmap revealed that the significant difference clades of the microbiome between the organic and inorganic Cu groups were grouped in cluster 1, while the specific clades in the CuCit group were grouped in cluster 2 (Figure 2D). The PCA analysis using these top 35 genera showed that the difference between organic and inorganic Cu groups could be explained by PC1, and the difference between CuCit and CuSO_4_ groups was explained by PC2 (Figure 2E).

**Figure 3:**
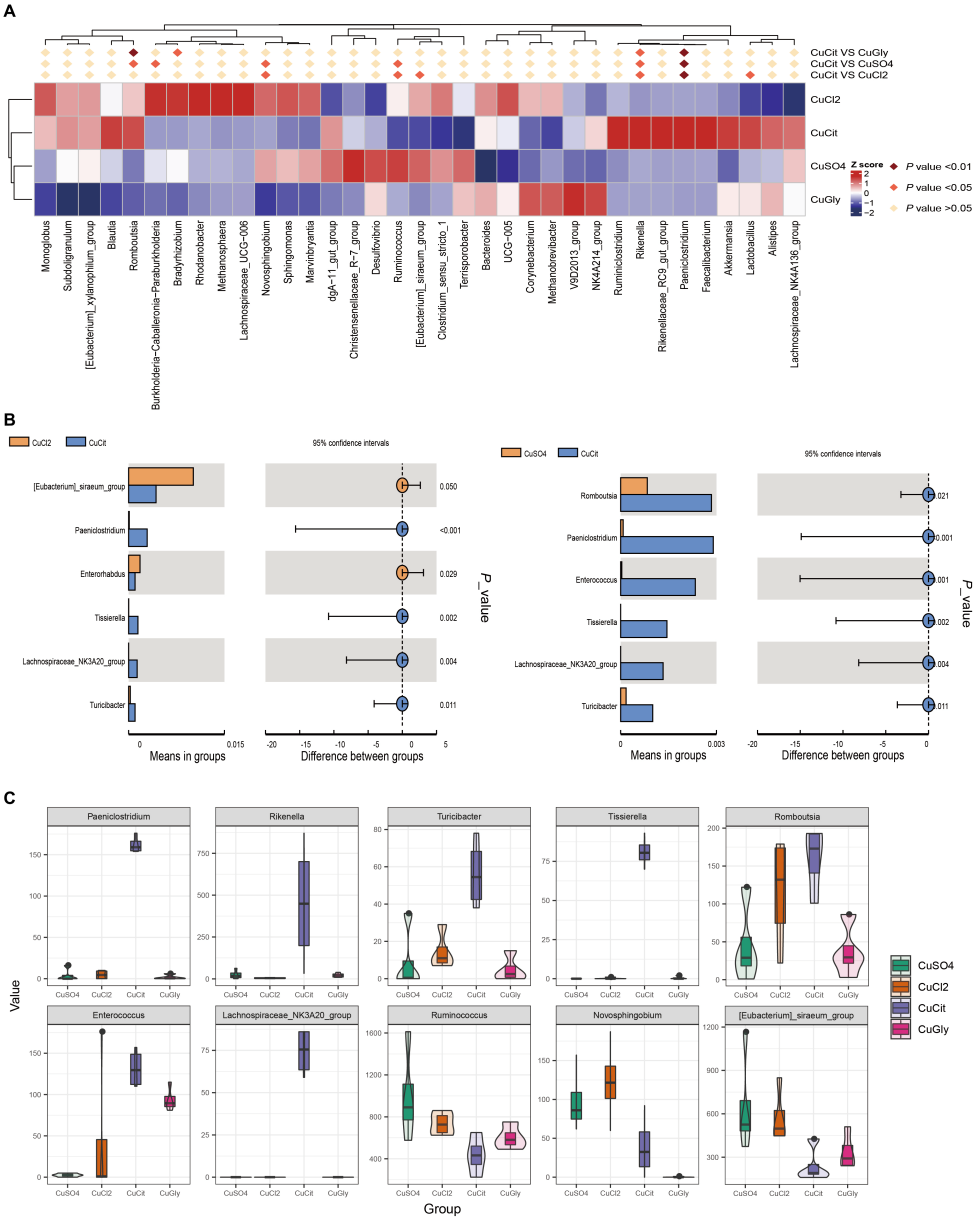
CuCit has significantly different microbial communities from the inorganic copper groups **(A)** MetaStat of differences at the genus level. **(B)** T-Test analysis of cupric citrate (CuCit) compared with two inorganic coppers. *p* < 0.05 indicated a statistically significant difference. **(C)** Box plot of the 10 different genera in the four groups.

In this study, we focused on the specific clades in the CuCit group to other groups. We found that the CuCit group had a significantly higher relative abundance of genera *Rikenella* and *Paeniclostridium* while the CuCit group had a significantly lower relative abundance of genera *Novosphingobium* and *Ruminococcus* (Figure 3A). The *t*-test analysis revealed that the CuCit group had significantly higher relative abundance of *Paeniclostridium*, *Tissierella*, *Lachnospiraceae_NK3A20_group*, *Enterococcus*, and *Turicibacter* than the CuCl_2_ group and CuSO_4_ group (*p* < 0.05) (Figures 3B,C).

### 3.3. Organic cu treatment decreased the SCFA production in the rabbit cecum

**Figure 4:**
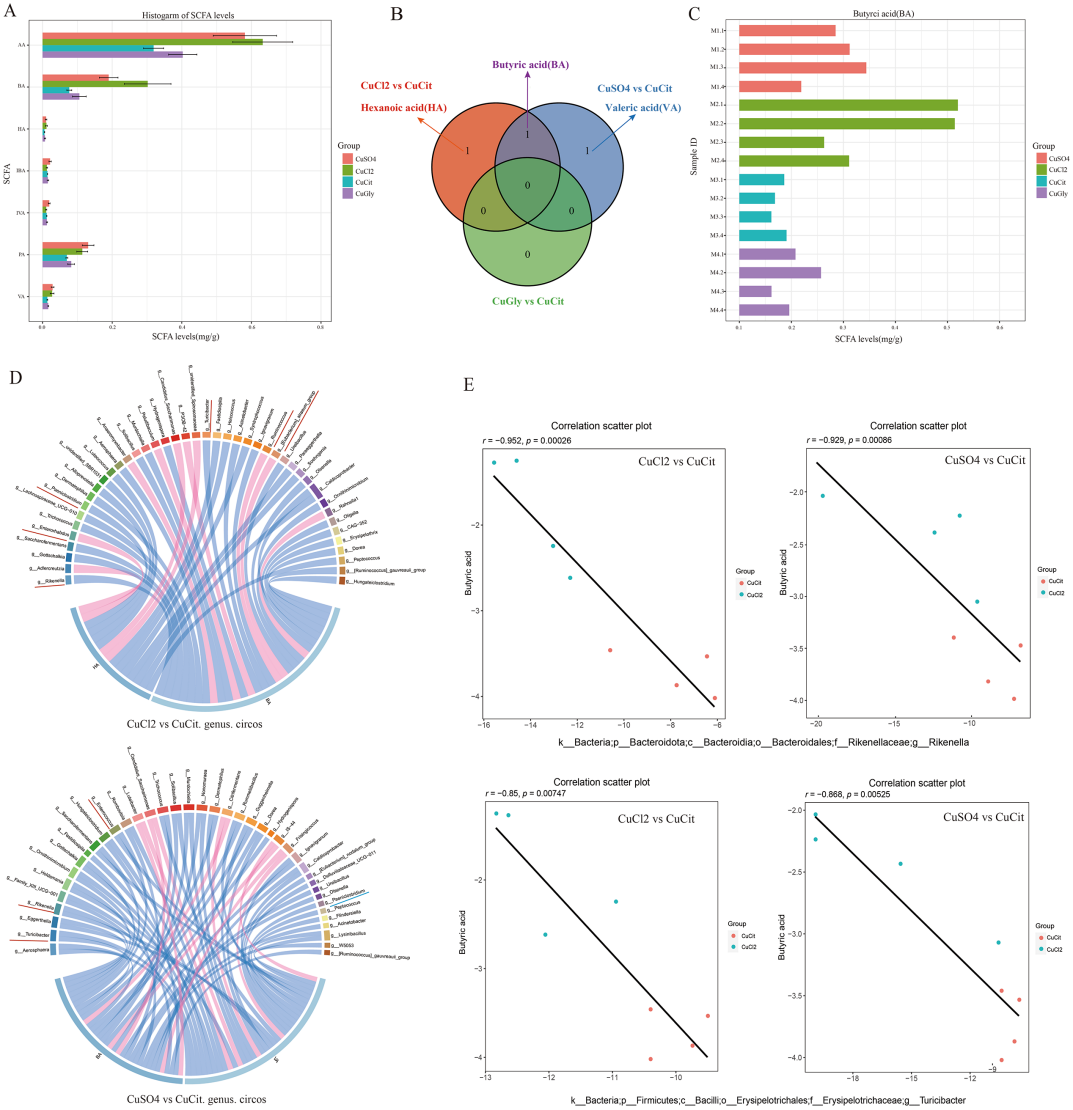
Short chain fatty acids(SCFAs) level and Spearman correlation between the butyric acid level and microbial composition in the cecum. **(A)** Histogram of SCFAs levels in different groups. **(B)** Venn of significantly different SCFAs between different groups. **(C)** Histogram of individual butyric acid (BA) levels. **(D)** Spearman correlation and circos plot of differential microbial genus abundance and differential SCFAs. **(E)** Scatter plot of Spearman correlation between microbial composition and butyric acid level.

The changes in the microbiota composition have been shown to alter the levels of bacterial metabolites in mice (17). We measured the effect of the different Cu source treatments on the levels of SCFA metabolites in the rabbit cecum. The levels of seven SCFAs in these four different Cu sources are shown in Figure 4A. The levels of acetic acid (AA), butyric acid (BA), propionic acid (PA), valeric acid (VA), and hexanoic acid (HA) were lower in the organic Cu groups than in the inorganic Cu groups (Figure 4A). The order of SCFAs level was AA followed by BA, and PA. The BA and HA levels significantly decreased in the CuCit group than in the CuCl_2_ group, and the BA and VA levels also significantly decreased in the CuCit group than in the CuSO_4_ group (Figure 4B). The individual BA levels are shown in Figure 4C. These results revealed that the level of BA significantly decreased in the organic Cu groups than in the inorganic Cu groups.

Then, the Spearman correlation of the microbial composition with SCFA metabolites was analyzed (Figures 4D,E). At the genus level, the relative abundance of *Rikenella* and *Turicibacter* was significantly negatively correlated with the BA level in the CuCit versus CuCl_2_ groups and CuCit versus CuSO_4_ groups (Figures 4D,E). The relative abundance of genus *Paeniclostridium* was significantly negatively correlated with the HA and VA levels in the CuCit versus CuCl_2_ groups and CuCit versus CuSO_4_ groups, respectively (Figure 4D). These results revealed that the CuCit group had an increased abundance of *Rikenella* and *Turicibacter* and a decreased BA level in the rabbit cecum.

## 4. Discussion

Our previous study has shown that rabbits fed with CuCit diets had a lower incidence of diarrhea compared with those treated with other Cu source diets (8). Adding CuCit to feed could effectively reduce the diarrhea rate of piglets (22).The effect of CuCit on reducing animal diarrhea may be due to the extensive antibacterial properties of copper. Adding copper to feed can regulate intestinal flora and affect physiological functions (23), while copper from different sources has different effects on gut structure and microbiota (4). In this study, we found that CuCit had a specific effect on cecal microbiota composition and SCFAs in rabbits.

The important finding of this study was that the CuCit group had a unique higher relative abundance of genera *Rikenella*, *Paeniclostridium*, *Tissierella*, *Turicibacter*, *Enterococcus*, and *Lachnospiraceae_NK3A20_group* compared with the other groups. However, the relative abundance of the genera *Novosphingobium* and *Ruminococcus* decreased in the organic Cu compared with the inorganic Cu groups. *Rikenella* belongs to the phylum *Bacteroidota*. *Bacteroidota* is an important part of the mammalian intestinal flora, capable of breaking down polysaccharides and proteins in the feed, which promotes the development of the intestinal immune system (24). *Rikenella* is a great anti diarrhea probiotics, and it is negatively correlated with diarrhea index (25). This is consistent with our experimental results. In the previous experimental results, the diarrhea rate of meat rabbits in the CuCit group was reduced by 55.57, 55.57, and 50.03% compared to the CuSO_4_, CuCl_2_ and CuGly group (8). The significant decrease in diarrhea rate of rabbits in the CuCit group may be related to the increase of *Rikenella*. *Enterococcus*, *Lachnospiraceae_NK3A20_group*, and *Ruminococcus* belong to the phylum *Firmicutes*. A previous study showed that *Lachnospiraceae_NK3A20_group* might participate in the metabolism of amino acids and glycerophospholipids, enhance antioxidant capacity, and promote the digestion and absorption of nutrients (26). There are also reports that compared to healthy individuals, the content of *Lachnospiraceae* in patients with colitis is lower (27). A report also showed that the addition of active tripeptides extracted from egg white can reduce diarrhea caused by *E. coli*, reduce the expression of inflammatory factors, increase the abundance of probiotics such as *Lachnospiraceae*, and reduce the abundance of pathogenic bacteria (28). Thus, the CuCit group having the unique higher relative abundance of genera *Rikenella*, and *Lachnospiraceae_NK3A20_group* might be beneficial to the lower incidence of diarrhea in rabbits. *Enterococcus* plays a protective role in regulating colonic homeostasis during development, inhibiting pathogen-mediated inflammatory responses in human intestinal epithelial cells, inducing interleukin-10 expression, and reducing the secretion of pro-inflammatory cytokines (29). *Ruminococcus* is a major fiber-degrading bacterium that significantly alters the digestion and utilization of nutrients in the gut (30). The increase in the abundance of *Ruminococcus* was positively correlated with the expression of Toll-like receptor genes (31), these genes are induced by host’s outer wall bacteria and bacterial products to activate the immune response to maintain homeostasis. The above results indicate that the regulation of gut microbiota may be an important factor in the anti diarrhea effect.

A phenomenon worthy of attention was that the BA level in the CuCit group was significantly lower than that in the inorganic Cu group. SCFAs played an important role in providing energy, regulating intestinal permeability, and inhibiting inflammation (32). AA, PA, and BA are important metabolites produced during intestinal microbial fermentation (33). The BA level is an important source of intestinal epithelial cells that maintain intestinal homeostasis (34). It is generally believed that the increase in the BA level is conducive to the balance of gut microbiota (35, 36). However, high concentrations of SCFAs are more likely to have the opposite effect, possibly increasing intestinal permeability and causing diarrhea (37). It has been reported that SCFAs can promote intestinal mucosal cells to release 5-hydroxy tryptamine (5-HT), thereby increasing intracellular Ca^2+^, activating K^+^channels to induce hyperpolarization, accelerating the contraction of intestinal smooth muscle, and excreting feces faster (38). When feed quickly passes through the intestine before water is absorbed by the large intestine, there is more water in the feces. In this experiment, SCFAs were significantly reduced in the CuCit group, which may lead to slower fecal passage and dryness, which helps alleviate diarrhea. The decrease in SCFAs in the CuCit group may be due to changes in gut microbiota. In this experiment, the BA levels in the CuCit group were negatively correlated with the relative abundance of *Rikenella* and *Turiciactor*. *Rikenella* ferments propionic acid in normal cells to generate energy and promote gluconeogenesi (39). It has been reported that a positive correlation between *Turiciactor* and BA (40). This is contrary to our results. In addition, *Lachnospiraceae* and *Ruminococcus* are also the important bacteria that produce butyric acid (30, 41). However, the abundance of *Lachnospiraceae* in the CuCit group increased while the abundance of *Ruminococcus* decreased, which may be due to the greater impact of *Ruminococcus* on butyric acid, leading to a decrease in butyric acid. On the other hand, SCFAs can regulate the morphological structure of the intestine and affect the digestion and absorption of nutrients (42). The decrease in SCFAs may be due to their absorption by intestinal epithelial cells, promoting the development of intestinal epithelial cells. The specific mechanism needs further exploration.

## 5. Conclusion

In summary, this study found that organic Cu and inorganic Cu sources had different effects on cecal microbiota composition and SCFAs in rabbits. The CuCit group had the unique higher relative abundance of genera *Rikenell* and *Lachnospiraceae_NK3A20_group*, which might be beneficial to the lower incidence of diarrhea in rabbits. In this experiment, we recommend a copper content of 20 mg/kg for CuCit, and we can further explore the effects of different concentrations of CuCit on intestinal microbiota and SCFAs to find the most suitable amount of CuCit added to the diet in rabbits.

## Data availability statement

The datasets presented in this study can be found in online repositories. The names of the repository/repositories and accession number(s) can be found in the article/supplementary material.

## Ethics statement

The animal study was reviewed and approved by the Institutional Animal Care and Use Committee of the Sichuan Animal Science Academy.

## Author contributions

JY and G-WZ designed the experiments. JY supervised the research work. G-WZ, YD, and YT analyzed the data. ZZ and RH assisted in sample collection. G-WZ and YD wrote the manuscript. JY revised the manuscript. All authors contributed to the article and approved the submitted version.

## Funding

This study was funded by the Sichuan Province Science and Technology Planning Project (2022ZHYZ0004).

## Conflict of interest

The authors declare that the research was conducted in the absence of any commercial or financial relationships that could be construed as a potential conflict of interest.

## Publisher’s note

All claims expressed in this article are solely those of the authors and do not necessarily represent those of their affiliated organizations, or those of the publisher, the editors and the reviewers. Any product that may be evaluated in this article, or claim that may be made by its manufacturer, is not guaranteed or endorsed by the publisher.
